# A Nomogram Model to Predict Early Recurrence of Patients With Intrahepatic Cholangiocarcinoma for Adjuvant Chemotherapy Guidance: A Multi-Institutional Analysis

**DOI:** 10.3389/fonc.2022.896764

**Published:** 2022-06-23

**Authors:** Qi Li, Jian Zhang, Chen Chen, Tianqiang Song, Yinghe Qiu, Xianhai Mao, Hong Wu, Yu He, Zhangjun Cheng, Wenlong Zhai, Jingdong Li, Dong Zhang, Zhimin Geng, Zhaohui Tang

**Affiliations:** ^1^ Department of Hepatobiliary Surgery, The First Affiliated Hospital of Xi’an Jiaotong University, Xi’an, China; ^2^ Department of Hepatobiliary Oncology, Tianjin Medical University Cancer Hospital, Tianjin, China; ^3^ Department of Biliary Surgery, Oriental Hepatobiliary Hospital Affiliated to Naval Medical University, Shanghai, China; ^4^ Department of Hepatobiliary Surgery, Hunan Provincial People’s Hospital, Changsha, China; ^5^ Department of Hepatobiliary and Pancreatic Surgery, West China Hospital of Sichuan University, Chengdu, China; ^6^ Department of Hepatobiliary Surgery, The First Hospital Affiliated to Army Medical University, Chongqing, China; ^7^ Department of Hepatobiliary Surgery, Zhongda Hospital of Southeast University, Nanjing, China; ^8^ Hepatobiliary Pancreas and Liver Transplantation Surgery, The First Affiliated Hospital of Zhengzhou University, Zhengzhou, China; ^9^ Department of Hepatobiliary Surgery, Affiliated Hospital of North Sichuan Medical College, Nanchong, China; ^10^ Department of General Surgery, Xinhua Hospital Affiliated to Shanghai Jiaotong University School of Medicine, Shanghai, China

**Keywords:** intrahepatic cholangiocarcinoma, recurrence, prognosis, nomogram, adjuvant chemotherapy

## Abstract

**Background:**

The influence of different postoperative recurrence times on the efficacy of adjuvant chemotherapy (ACT) for intrahepatic cholangiocarcinoma (ICC) remains unclear. This study aimed to investigate the independent risk factors and establish a nomogram prediction model of early recurrence (recurrence within 1 year) to screen patients with ICC for ACT.

**Methods:**

Data from 310 ICC patients who underwent radical resection between 2010 and 2018 at eight Chinese tertiary hospitals were used to analyze the risk factors and establish a nomogram model to predict early recurrence. External validation was conducted on 134 patients at the other two Chinese tertiary hospitals. Overall survival (OS) and relapse-free survival (RFS) were estimated by the Kaplan–Meier method. Multivariate analysis was conducted to identify independent risk factors for prognosis. A logistic regression model was used to screen independent risk variables for early recurrence. A nomogram model was established based on the above independent risk variables to predict early recurrence.

**Results:**

ACT was a prognostic factor and an independent affecting factor for OS and RFS of patients with ICC after radical resection (*p* < 0.01). The median OS of ICC patients with non-ACT and ACT was 14.0 and 15.0 months, and the median RFS was 6.0 and 8.0 months for the early recurrence group, respectively (*p* > 0.05). While the median OS of ICC patients with non-ACT and ACT was 41.0 and 84.0 months, the median RFS was 20.0 and 45.0 months for the late recurrence group, respectively (*p* < 0.01). CA19-9, tumor size, major vascular invasion, microvascular invasion, and N stage were the independent risk factors of early recurrence for ICC patients after radical resection. The C-index of the nomogram was 0.777 (95% CI: 0.713~0.841) and 0.716 (95%CI: 0.604~0.828) in the training and testing sets, respectively.

**Conclusion:**

The nomogram model established based on the independent risk variables of early recurrence for curatively resected ICC patients has a good prediction ability and can be used to screen patients who benefited from ACT.

## Introduction

Intrahepatic cholangiocarcinoma (ICC) is the second most common primary liver cancer and accounts for about 10% to 15% (1, 2). Over the past two decades, the incidence of ICC has been increasing throughout the world, also accompanied by an increase in mortality (3, 4). At present, surgical resection is considered the only curative treatment for ICC patients, but only a small fraction (15%) of patients are eligible for surgery (5). The occurrence of postoperative recurrence and metastasis leads to poor survival even after curative hepatectomy, with a 3-year relapse-free survival (RFS) rate below 30% and a 5-year overall survival (OS) rate ranging from 20% to 40% (6–8). Therefore, identifying patients who are at risk for early recurrence is important to construct individualized surveillance strategies for ICC patients after radical resection. Recently, more and more scholars are concerned about the risk factors of early recurrence, while the definition of early recurrence is different because definitive guidelines do not exist (9–11).

Currently, the effect of adjuvant chemotherapy (ACT) on the prognosis of ICC patients is still controversial (12–14), although studies have proved that ACT can improve the prognosis (15, 16). Because many factors may affect the efficacy of ACT, it is very important to screen potential patients who could benefit from ACT. Few published studies focused on the recurrence time of ICC patients accompanied by ACT or not, which to some extent could help choose patient groups that are suitable for ACT. This study aimed to investigate the independent risk factors and establish a nomogram model to predict early recurrence to screen ICC patients for ACT.

## Material and Methods

### Patients

All patients undergoing curative resection for histologically confirmed ICC between 2010 and 2018 at ten tertiary hospitals in China (Tianjin Medical University Cancer Institute and Hospital; Hunan Provincial People’s Hospital; The First Hospital Affiliated to Army Medical University; The First Affiliated Hospital of Xi’an Jiaotong University; Zhongda Hospital of Southeast University; The First Affiliated Hospital of Zhengzhou University; Xinhua Hospital Affiliated to Shanghai Jiaotong University School of Medicine; Affiliated Hospital of North Sichuan Medical College; Oriental Hepatobiliary Hospital Affiliated to Naval Medical University; West China Hospital of Sichuan University) were considered for inclusion. The study was approved by the ethics committee of Xinhua Hospital Affiliated to Shanghai Jiaotong University School of Medicine (No. XHEC-JDYXY-2018-002), Shanghai, China, as well as by the ethics committees of the other centers. Written informed consent was obtained from all included patients and their families before study enrollment.

According to previous studies (10, 17, 18), a postoperative recurrence within 1 year was defined as early recurrence, while a recurrence of >1 year was a late recurrence. The inclusion criteria were as follows (1): patients underwent radical resection and the margin status of the initial resection was microscopically negative (R0) (2); patients had a detailed postoperative recurrence record (3); patients received ACT with complete and systematic regimens; and (4) patients without a history of other malignancies. Exclusion criteria were as follows (1): hilar cholangiocarcinoma invading the liver (2); mixed cholangiocarcinoma-hepatocellular carcinoma (3); incomplete clinical data; and (4) patients died within 30 days after surgery.

### The Regimens and Indications of ACT

In this study, patients with ACT were strictly performed as follows. The regimens included gemcitabine (1,000 mg/m^2^ on days 1 and 8) + capecitabine (1,250 mg/m^2^ twice daily on days 1–14) of a 3-week cycle; gemcitabine (1,000 mg/m^2^ on days 1 and 8) + cisplatin (30 mg/m^2^ on days 1 and 8) of a 3-week cycle; gemcitabine (1,000 mg/m^2^ on days 1 and 8) + oxaliplatin (100 mg/m^2^ on day 1) of a 3-week cycle; gemcitabine (1,000 mg/m^2^ on days 1 and 8) + tegafur (40~60 mg twice daily on days 1–14) of a 3-week cycle.

The indications for ACT were ICC patients with T2~4 stage, N1 stage, combined with major vascular invasion, microvascular invasion, perineural invasion, etc., which was associated with high postoperative recurrence risk.

### Follow-up

Follow-up was performed in outpatient or telephone. Liver function, tumor biomarkers, ultrasound, contrast-enhanced CT, or MRI examinations were reviewed every 2 to 3 months within 1 year after surgery, and then once every 3–6 months for more than 1 year after surgery. Postoperative recurrence was defined as the discovery of new lesions by two or more imaging examinations. All included patients were followed up through December 2020.

### Statistical Analysis

All statistical analyses were performed using SPSS version 25 (IBM Corp., Armonk, NY, USA). Continuous variables were expressed as the mean ± standard deviation. Categorical variables were examined using the *χ* ^2^-test. The Kaplan–Meier method and Log-rank test were conducted for univariate analysis, and the Cox proportional hazard regression model was conducted for multivariate analysis. A logistic regression model was further used to screen independent risk variables for early recurrence. Survival analysis curves were conducted by GraphPad Prism (version 8.0, San Diego, California, USA). *p* < 0.05 was considered statistically significant.

### Development and Assessment of the Nomogram

A total of 444 ICC patients were finally included in the study; 310 patients from 8 medical centers were included as the training set, and 134 patients from the Oriental Hepatobiliary Hospital Affiliated to Naval Medical University and West China Hospital of Sichuan University were included as the testing set. R software version 3.6.1 (http://www.r-project.org/) was used to produce a nomogram prediction model based on the independent risk variables for early recurrence of ICC patients after surgery. The performance of the nomogram was evaluated based on the concordance index (C-index), calibration plot, and decision curve analysis (DCA). DCA was performed by calculating the benefit of a series of threshold probabilities and was conducted to evaluate the clinical practicability of the nomogram (19).

## Results

A total of 444 patients undergoing radical resection for histologically confirmed ICC between 2010 and 2018 were considered for inclusion. The 1-, 3-, and 5-year OS rates of patients were 80.9%, 40.4%, and 19.4%, and the 1-, 3-, and 5-year RFS rates of patients were 55.5%, 17.4%, and 13.3%, respectively. Median survival time was 26.0 and 14.8 months for OS and RFS in the training dataset, respectively.

### Survival Analysis Between Early Recurrence and Late Recurrence Groups

**Figure 1:**
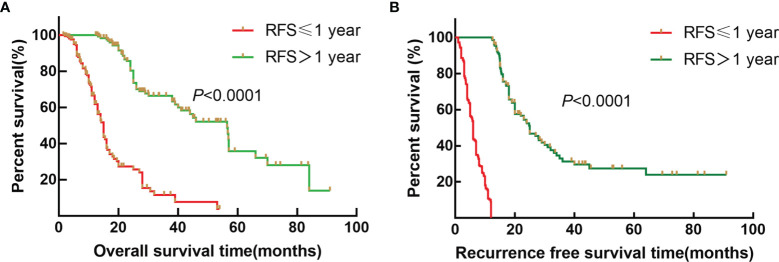
Survival curves of patients with ICC after radical resection between the early recurrence group and late recurrence group. **(A)** Kaplan–Meier OS curve for ICC patients between the early recurrence group and late recurrence group. **(B)** Kaplan–Meier RFS curve for ICC patients between the early recurrence group and late recurrence group.

To further explore the survival difference between early recurrence and late recurrence groups, the results showed that early recurrence (HR: 6.585, 95% CI:4.454~9.736) was a risk factor for OS of ICC patients after radical resection compared to the late recurrence group (*p* < 0.001). Furthermore, the median OS was 15.0 and 56.5 months (**Figure 1A**, *p* < 0.001), and the median RFS were 7.0 and 15.0 months for early recurrence and late recurrence groups of ICC patients, respectively (**Figure 1B**, *p* < 0.001). Therefore, the results showed that early recurrence was an adverse factor for the prognosis of ICC after radical resection.

### Survival Analysis of ICC Patients Between Non-ACT and ACT for the Early Recurrence and Late Recurrence Groups

To determine whether the ACT regimens affected the prognosis of patients, we first analyzed the prognosis differences among the four regimens for patients treated with ACT. The results showed that there was no difference in OS and RFS among different chemotherapy regimens (*p* > 0.05). Univariate analysis then showed that ACT (HR: 0.523, 95% CI:0.364~0.753; HR: 0.653, 95% CI:0.488~0.875) was the prognostic factor for OS and RFS of patients with ICC after radical resection (*p* < 0.01). Multivariate analysis showed that ACT (HR: 0.403, 95% CI: 0.269~0.603; HR: 0.672, 95% CI: 0.502~0.900) was an independent prognostic factor for OS and RFS (*p* < 0.01) (**Table 1**).

**Table 1:** Univariate and multivariate analyses of prognosis for ICC after radical resection.

	OS				RFS			
	Univariate analysis		Multivariate analysis		Univariate analysis		Multivariate analysis	
	HR (95% CI)	*p*-value	HR (95% CI)	*p*-value	HR (95% CI)	*p*-value	HR (95% CI)	*p*-value
Sex								
Female vs. male	0.792 (0.567~1.106)	0.170			0.748(0.566~1.088)	0.061		
Age (year)								
>55 vs. ≤55	1.153 (0.869~1.769)	0.070			0.978(0.739~1.295)	0.876		
Obstructive jaundice								
Yes vs. no	1.193 (0.756~1.882)	0.449			1.046 (0.701~1.560)	0.827		
HBV infection								
Yes vs. no	0.590 (0.387~0.899)	0.014			1.052 (0.764~1.449)	0.755		
Hepatolithiasis								
Yes vs. no	1.459 (1.022~2.081)	0.038			1.149 (0.845~1.563)	0.375		
AFP (ng/ml)								
>7.0 vs. ≤7.0	1.081 (0.742~1.576)	0.686			1.022 (0.750~1.391)	0.892		
CEA (ng/ml)								
>5.0 vs. ≤5.0	1.495 (1.036~2.158)	0.032			1.368 (1.011~1.851)	0.043		
CA19-9 (U/ml)								
>39.0 vs. ≤39.0	1.529 (1.148~1.864)	0.019	1.516 (1.117~1.959)	0.013	1.827 (1.367~2.585)	0.002	1.474 (1.104~1.968)	0.008
CA125 (U/ml)								
>35.0 vs. ≤35.0	1.490 (1.062~2.090)	0.021			1.427 (1.070~1.902)	0.016		
Child–Pugh grade								
Grade B vs. A	1.086 (0.661~1.785)	0.744			1.021 (0.666~1.566)	0.924		
Tumor differentiation								
Moderate vs. well	1.289 (1.020~2.212)	0.039			1.237 (1.089~1.990)	0.033		
Poor vs. well	1.815 (1.720~2.403)	0.013			1.998 (1.667~2.807)	0.014		
Tumor location								
Right vs. left	0.775 (0.541~1.108)	0.162			0.862 (0.641~1.159)	0.326		
Left and right vs. Left	0.838 (0.464~1.156)	0.560			0.693 (0.429~1.118)	0.693		
Morphologic grape								
Periductal infiltrating vs. mass-forming	0.108 (0.714~1.719)	0.647			1.298 (0.900~1.871)	0.162		
Intraductal growth vs. mass-forming	1.047 (0.561~1.953)	0.886			1.053 (0.645~1.722)	0.836		
Tumor size (cm)								
>5.0 vs. ≤5.0	1.293 (1.117~1.825)	0.030			2.147 (1.864~2.522)	0.003	1.734 (1.337~2.348)	0.008
Major vascular invasion								
Yes vs. no	1.670 (1.086~2.566)	0.019			1.504 (1.039~2.177)	0.030		
Microvascular invasion								
Yes vs. no	1.864 (1.338~2.586)	0.003	2.235 (1.338~3.733)	0.002	1.329 (1.013~1.935)	0.038		
Perineural invasion								
Yes vs. no	1.959 (1.287~2.981)	0.002	1.813 (1.344~2.444)	<0.001	1.551 (1.144~1.934)	0.010		
Liver capsule involvement								
Yes vs. no	1.014 (0.700~1.470)	0.939			1.202 (0.804~1.792)	0.365		
AJCC 8th edition T stage								
T_2_ vs. T_1a_/T_1b_	2.029 (1.273~3.233)	0.003			1.224 (1.013~2.149)	0.024		
T_3_/T_4_ vs. T_1a_/T_1b_	3.227 (1.826~5.701)	<0.001			1.974 (1.346~2.893)	<0.001		
AJCC 8th edition N stage								
N1 vs N0	2.076 (1.444~2.984)	<0.001	1.698 (1.158~2.488)	0.007	1.716 (1.275~2.310)	<0.001	1.618 (1.199~2.184)	0.002
AJCC 8th edition TNM stage								
II vs. IA/IB	1.213 (1.058~1.939)	0.021			1.287 (1.147~1.908)	0.013		
IIIA/IIIB/IV vs. IA/IB	1.707 (1.177~2.475)	0.005			1.878 (1.491~2.536)	<0.001		
Adjuvant chemotherapy								
Yes vs. no	0.523 (0.364~0.753)	<0.001	0.403 (0.269~0.603)	<0.001	0.653 (0.488~0.875)	0.004	0.672 (0.502~0.900)	0.008

**Figure 2:**
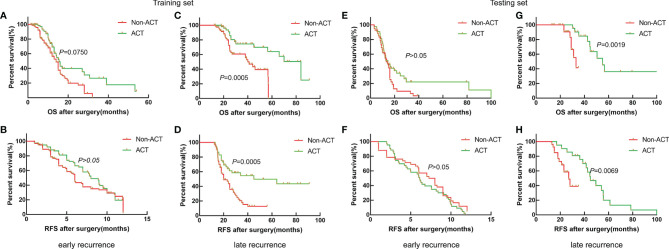
Survival curves of patients with ICC after radical resection between non-ACT and ACT for the early recurrence group and late recurrence group. **(A, B)** Kaplan–Meier OS curve and RFS curve for the early recurrence group in the training set. **(C, D)** Kaplan–Meier OS curve and RFS curve for the late recurrence group in the training set. **(E, F)** Kaplan–Meier OS curve and RFS curve for the early recurrence group in the testing set. **(G, H)** Kaplan–Meier OS curve and RFS curve for the late recurrence group in the testing set.

To further stratify analysis in the training set, the results also showed that the median OS of ICC patients with non-ACT and ACT was 14.0 and 15.0 months; the median RFS was 6.0 and 8. months for the early recurrence group, respectively (**Figures 2A, B**, *p* > 0.05); the median OS of ICC patients with non-ACT and ACT was 41.0 and 84.0 months, and the median RFS was 20.0 and 45.0 months for the late recurrence group, respectively (**Figures 2C, D**, *p* < 0.01).

Similarly, the results also showed that ACT could be beneficial to the late recurrence group for OS and RFS in the testing set (**Figures 2G, H**, *p* < 0.01), while the OS and RFS of ICC patients with early recurrence were not significantly improved after receiving ACT (**Figure 2E, F**, *p* > 0.05). Thus, the results showed that ACT can improve the prognosis for ICC patients with late recurrence significantly.

### Development of the Nomogram Prediction Model

**Figure 3:**
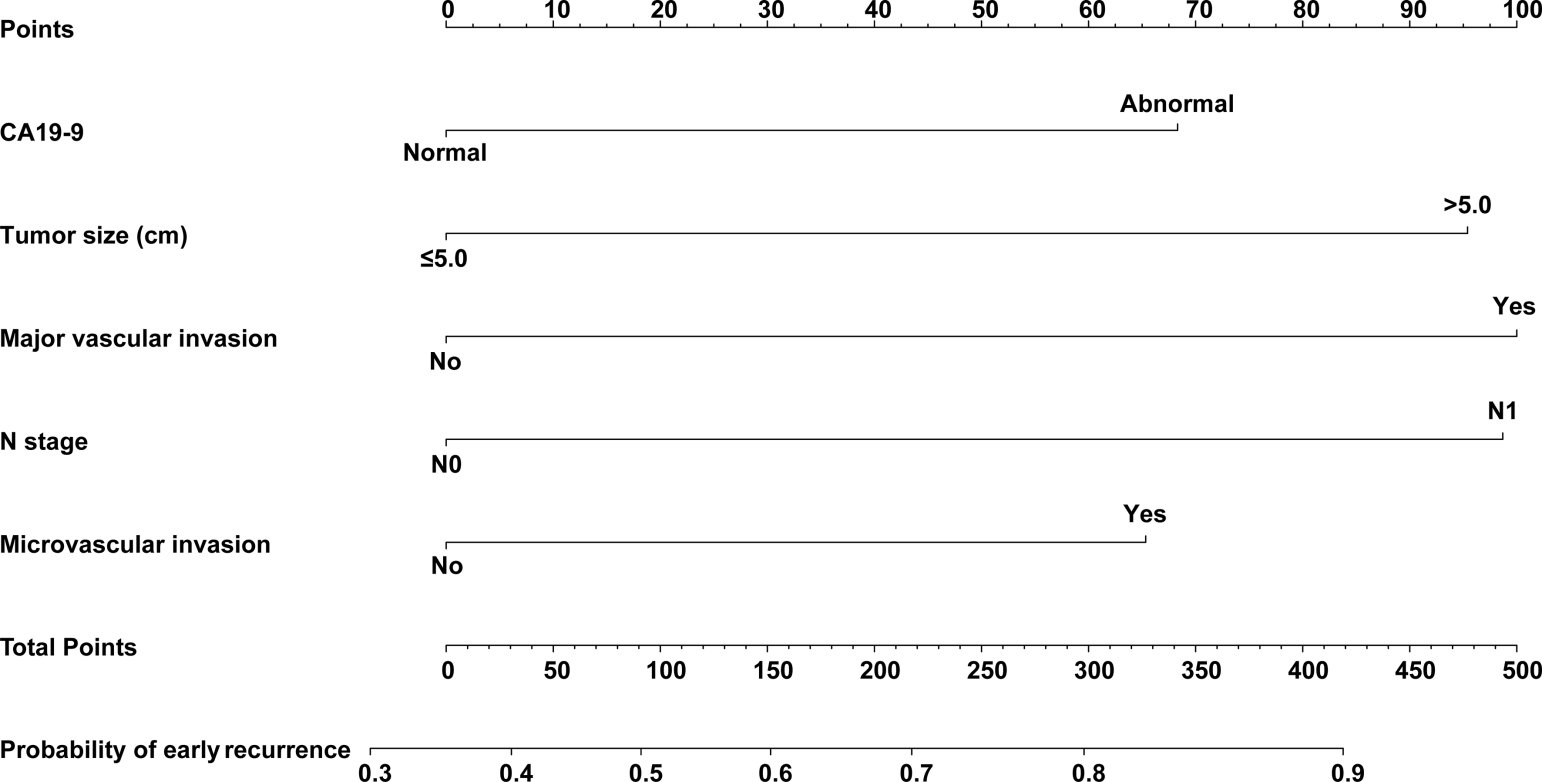
Nomogram prediction model for predicting early recurrence of patients with ICC after radical resection.

CA19-9, tumor size, major vascular invasion, microvascular invasion, and N stage were the independent risk factors for early recurrence of ICC patients after radical resection. A nomogram to predict early recurrence was established based on the above independent risk factors. Detailed results of the logistic regression are shown on the right-hand side of **Table 2**. The nomogram is shown in **Figure 3**, and an online calculator for the nomogram model was established, which is available at https://doczj.shinyapps.io/icc_early.

**Table 2:** Comparison of clinicopathologic characteristics of early recurrence and late recurrence for ICC after radical resection.

	Early recurrence group	Late recurrence group	Univariate analysis		Multivariate analysis	
	No. (%)	No. (%)	*χ* ^2^	*p*-value	HR (95% CI)	*p*-value
Sex						
Male	98 (55.4)	67 (50.4)	0.760	0.383		
Female	79 (44.6)	66 (49.6)				
Age (year)						
≤55	71 (40.1)	56 (42.1)	0.125	0.724		
>55	106 (59.9)	77 (57.9)				
Obstructive jaundice						
No	159 (89.8)	115 (86.5)	0.837	0.360		
Yes	18 (10.2)	18 (13.5)				
HBV infection						
No	140 (79.1)	97 (72.9)	1.603	0.206		
Yes	37 (20.9)	36 (27.1)				
Hepatolithiasis						
No	135 (76.3)	98 (73.7)	0.272	0.602		
Yes	42 (23.7)	35 (26.3)				
AFP (ng/ml)						
≤7.0	137 (77.4)	91 (68.4)	3.148	0.076		
>7.0	40 (22.6)	42 (31.6)				
CEA (ng/ml)						
≤5.0	119 (67.2)	105 (78.9)	5.200	0.023		
>5.0	58 (32.8)	28 (21.1)				
CA19-9 (U/ml)						
≤39.0	61 (34.5)	64 (48.1)	5.886	0.015	1.624 (1.005~2.62)	0.048
>39.0	116 (65.5)	69 (51.9)				
CA125 (U/ml)						
≤35.0	104 (58.8)	91 (68.4)	3.039	0.081		
>35.0	73 (41.2)	42 (31.6)				
Child–Pugh grade						
A	163 (92.1)	117 (88.0)	1.475	0.225		
B	14 (7.9)	16 (12.0)				
Tumor differentiation						
Well	10 (5.6)	16 (12.0)	4.498	0.106		
Moderate	112 (63.3)	83 (62.4)				
Poor	55 (31.1)	34 (25.6)				
Tumor location						
Left	92 (52.0)	62 (46.6)	1.881	0.390		
Right	68 (38.4)	52 (39.1)				
Both	17 (9.6)	19 (14.3)				
Morphologic grape						
Mass-forming	140 (79.1)	103 (77.4)	2.862	0.239		
Periductal infiltrating	28 (15.8)	17 (12.8)				
Intraductal growth	9 (5.1)	13 (9.8)				
Tumor size (cm)						
≤5.0	84 (47.5)	89 (66.9)	11.660	0.001	2.239 (1.405~3.570)	0.001
>5.0	93 (52.5)	44 (33.1)				
Major vascular invasion						
No	142 (80.2)	121 (91.0)	6.824	0.009	2.485 (1.235~5.000)	0.011
Yes	35 (19.8)	12 (9.0)				
Microvascular invasion						
No	139 (78.5)	118 (88.7)	5.564	0.018	2.151 (1.127~4.103)	0.020
Yes	38 (21.5)	15 (11.3)				
Perineural invasion						
No	136 (76.8)	114 (85.7)	3.835	0.050		
Yes	41 (23.2)	19 (14.3)				
Liver capsule involvement						
No	124 (70.1)	102 (76.7)	1.692	0.193		
Yes	53 (29.9)	31 (23.3)				
AJCC 8th edition T stage						
T_1a_/T_1b_	35 (19.8)	52 (39.1)	14.919	0.001		
T_2_	104 (58.8)	64 (38.1)				
T_3_/T_4_	38 (21.5)	17 (12.8)				
AJCC 8th edition N stage						
N0	108 (61.0)	106 (79.7)	12.398	<0.001	2.225 (1.303~3.801)	0.003
N1	69 (39.0)	27 (20.3)				
AJCC 8th edition TNM stage						
IA/IB	69 (39.0)	78 (58.6)	13.451	0.001		
II	30 (12.9)	21 (15.8)				
IIIA/IIIB/IV	78 (44.1)	34 (25.6)				
Adjuvant chemotherapy						
No	100 (56.5)	80 (60.2)	0.416	0.519		
Yes	77 (43.5)	53 (39.8)				

### Assessment of the Nomogram Prediction Model

**Figure 4:**
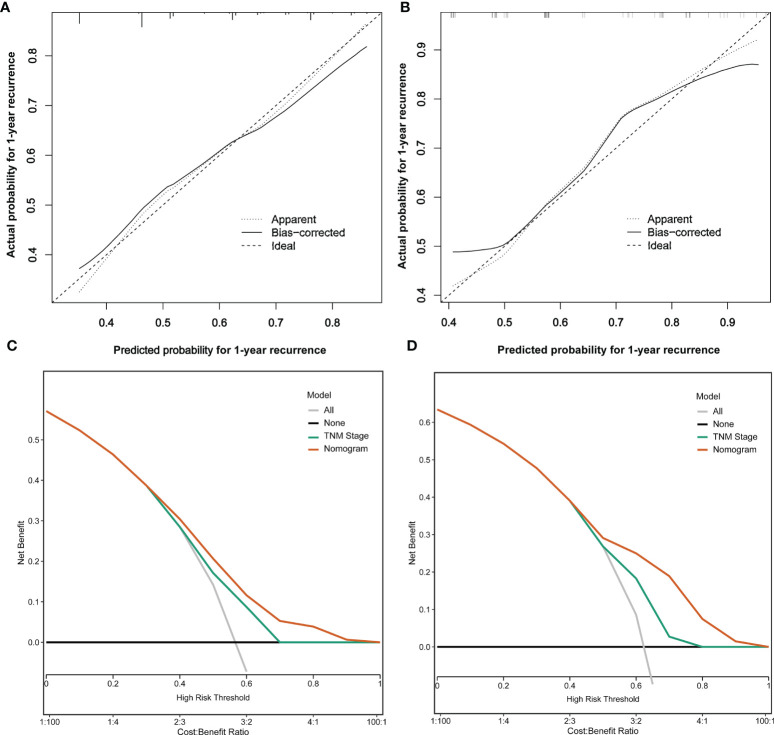
Analysis of calibration plots and decision curves for the nomogram prediction model. **(A)** Calibration plot for the nomogram in the training set. **(B)** Calibration plot for the nomogram in the testing set. **(C)** Decision curve analysis for the nomogram in the training set. **(D)** Decision curve analysis for the RFS in the testing set.

The C-index of the nomogram model was 0.777 (95% CI: 0.713~0.841) and 0.716 (95% CI: 0.604~0.828) in the training and testing sets, respectively. The calibration plots are shown in **Figures 4A, B**, which showed the prediction results were more consistent with the actual results. In addition, DCAs are shown in **Figures 4C, D**, which showed that the predictive ability of the nomogram model was better than TNM staging in the training set and testing set.

## Discussion

Guidelines for the management of recurrent ICC remain controversial and poorly defined, and a few studies have analyzed the risk factors of early recurrence with different criteria. Tsilimigras et al. (9) analyzed the risk factors of very early recurrence (≤6 months) and developed an easy-to-use online calculator to help clinicians predict the chance of very early recurrence, which provided treatment and surveillance strategies for ICC patients after surgery. Zhang et al. (11) revealed that the patterns of early recurrence (≤2 years) and late recurrence were different, and early recurrence of extrahepatic recurrence was more common, whereas late recurrence was often only intrahepatic recurrence. Importantly, patients’ recurrence within 1 year after surgery may represent more aggressive tumor biology, and a cutoff of 1 year after surgery has been used to distinguish the early recurrence and late recurrence in most studies (10, 17, 20, 21). Wang et al. (20) showed that specific risk factors, including CA 19-9, microvascular invasion, and multiple tumors, may relate to the early recurrence of ICC after curative resection. Xing et al. (22) revealed that CA 19-9, tumor number at recurrence, and treatment for recurrence could be used to assess survival for post-operative recurrence, and time to recurrence, especially within a year after resection, had a significant impact on postrecurrence survival. In this study, CA19-9, tumor size, major vascular invasion, microvascular invasion, and N stage were identified as the independent risk factors for early recurrence, in which CA19-9 and N stage were the independent risk factors for OS and RFS of ICC patients after radical resection. Many studies (11, 17, 20, 23–25) have proved that the above five variables were the independent risk factors for early recurrence and prognosis, which also provided a basis for establishing an effective predictive model.

Many studies (15, 16, 26) revealed that ACT was beneficial to ICC patients after radical resection, but which patients were suitable for ACT also required further study. In this study, ACT was also a protective prognostic factor for ICC patients in the late recurrence group. Unfortunately, patients in the early recurrence group did not benefit from ACT. Hence, ACT seemed to have less benefit for curatively resected ICC patients when analyzed as a complete group. However, an obvious survival benefit was shown when all patients were divided into early and late recurrence groups (27, 28).

Moreover, early recurrence assessment could provide references for repeat hepatic resection to produce long-term survival outcomes in previous studies (10, 17, 29). Therefore, an accurate prediction of early recurrence was of great value for appropriate treatment strategies for ICC patients after surgery, particularly because this study identified that ACT would not benefit patients at a high risk of early recurrence. In addition, the exploration of an effective treatment to improve prognosis is of great importance for early recurrence patients.

To our knowledge, this study is the first to establish a nomogram prediction model for early recurrence including the above independent risk variables. The C-index of the nomogram model was 0.777 and 0.716 in the training and testing sets, respectively. The calibration plots showed that the prediction results were more consistent with the actual results, and DCAs showed that the predictive ability of the nomogram model was better than TNM staging in the training and testing sets. Different from our nomogram model, many scholars (21, 30, 31) developed radiomics nomograms by using the radiomics signature and other clinicopathological characteristics to predict the early recurrence of ICC after surgery, but the inclusion of radiomics signature also brought certain difficulties to clinical applications. Jeong et al. (32) established a nomogram model to allow precise estimation of the risk of 1-, 3-, and 5-year RFS for ICC after resection by the combined Cox and logistic ranking system based on 10 and 11 covariates; however, it could not evaluate and predict early recurrence and was complex in the application despite its good predictive ability. Yu et al. (33) established a nomogram for 1-, 3-, and 5-year RFS based on tumor size, tumor number, direct invasion, and triosephosphate isomerase (TPI1), while they did not provide treatment decisions for postoperative early and late recurrence, although they showed the prognostic model was accurate in predicting recurrence for ICC patients. Therefore, our nomogram model has better clinical practicability and applicability for early recurrence of ICC patients by using the online calculator.

However, several limitations must be acknowledged in this study. It is difficult to avoid selection bias in the retrospective design and the different definitions of early recurrence. In addition, the reasons why ICC patients with early recurrence cannot benefit from ACT have not been further analyzed. Accordingly, we recommend that more patients with ICC after radical resection from other medical centers could be collected in future studies to validate our results, and the molecular biomarkers should be added into the study to improve the predictive ability of the nomogram model, which can provide decision support for ACT of ICC patients more effectively.

In summary, this study retrospectively analyzed 444 patients with ICC after radical resection and developed a nomogram prediction model based on the risk factors of early recurrence, including CA19-9, tumor size, major vascular invasion, microvascular invasion, and N stage with a good predictive ability, which can be used to screen patients with ICC who benefit from ACT effectively. We expect that the nomogram model can help to screen appropriate ICC patients who could benefit from ACT and achieve widespread clinical application in the future.

## Data Availability Statement

The raw data supporting the conclusions of this article will be made available by the authors, without undue reservation.

## Ethics Statement

The studies involving human participants were reviewed and approved by the ethics committee of Xinhua Hospital Affiliated to Shanghai Jiaotong University School of Medicine (No. XHEC-JDYXY-2018-002), Shanghai, China. The patients/participants provided their written informed consent to participate in this study.

## Author Contributions

ZG and ZT conceived and designed the experiments. QL and JZ performed the experiments. TS, YQ, XM, HW, YH, ZC, WZ, and JL collected and offered the data. QL and JZ contributed analysis tools. QL, JZ, CC, and DZ conducted statistical analysis. QL and JZ wrote the paper. ZG and ZT reviewed the manuscript. All authors read and approved the final manuscript.

## Funding

This study was supported by the National Natural Science Foundation of China (No. 62076194, No. 81772521); Multicenter Clinical Research Project of Shanghai Jiaotong University, School of Medicine (DLY201807); and Clinical Training Program of Shanghai Xinhua Hospital Affiliated to Shanghai Jiaotong University, School of Medicine (17CSK06).

## Conflict of Interest

The authors declare that the research was conducted in the absence of any commercial or financial relationships that could be construed as a potential conflict of interest.

## Publisher’s Note

All claims expressed in this article are solely those of the authors and do not necessarily represent those of their affiliated organizations, or those of the publisher, the editors and the reviewers. Any product that may be evaluated in this article, or claim that may be made by its manufacturer, is not guaranteed or endorsed by the publisher.
